# Larger Perioperative Opioid Prescriptions Lead to Prolonged Opioid Use After Hand and Upper Extremity Surgery: A Multicenter Analysis

**DOI:** 10.5435/JAAOSGlobal-D-22-00036

**Published:** 2022-10-24

**Authors:** Clay B. Townsend, Justin A. Ly, Ryan Judy, Matthew B. Sherman, Nick Elmer, Christine Conroy, Hesham M. Abdelfattah, Mark K. Solarz, Katharine Woozley, Asif M. Ilyas

**Affiliations:** From the Rothman Orthopaedic Institute (Dr. Townsend, Mr. Sherman, and Dr. Ilyas); the Lewis Katz School of Medicine, Temple University (Mr. Ly); Orthopaedic Surgery and Sports Medicine, Temple University Hospital (Dr. Judy, Dr. Abdelfattah, and Dr. Solarz); the Department of Orthopaedics, Einstein Healthcare Network (Dr. Conroy and Dr. Woozley); the Rothman Orthopaedic Institute Foundation for Opioid Research & Education (Dr. Ilyas); and the Sidney Kimmel Medical College, Thomas Jefferson University (Mr. Elmer and Dr. Ilyas), Philadelphia, PA.

## Abstract

The opioid epidemic remains an ongoing public health crisis. The purpose of this study was to investigate whether surgeons' prescribing patterns of the initial postoperative opioid prescription predispose patients to prolonged opioid use after upper extremity surgery.

**Methods:**

This multicenter retrospective study was done at three academic institutions. Patients who underwent carpal tunnel release, basal joint arthroplasty, and distal radius fracture open reduction and internal fixation over a 1.5-year period were included. Opioid prescription data were obtained from the Pennsylvania Prescription Drug Monitoring Program website.

**Results:**

Postoperatively, 30.1% of the patients (191/634) filled ≥1 additional opioid prescription, and 14.0% (89/634) experienced prolonged opioid use 3 to 6 months postoperatively. Patients who filled an additional prescription postoperatively were initially prescribed significantly more pills (*P* = 0.001), a significantly longer duration prescription (*P* = 0.009), and a significantly larger prescription in total milligram morphine equivalents (*P* = 0.002) than patients who did not fill additional prescriptions. Patients who had prolonged opioid use were prescribed a significantly longer duration prescription (*P* = 0.026) than those without prolonged use.

**Conclusion:**

Larger and longer duration of initial opioid prescriptions predisposed patients to continued postoperative opioid use. These findings emphasize the importance of safe and evidence-based prescribing practices to prevent the detrimental effects of opioid use after orthopaedic surgery.

The opioid epidemic remains an ongoing national public health crisis in the United States, with more than 11 million Americans reporting misusing prescription opioids annually.^1^ Even more harrowing, since 1999, drug overdose deaths involving an opioid have increased sixfold.^1^ This crisis has introduced immense financial burden on both patients suffering from opioid addiction and on the healthcare system as a whole.^2,3^ As some of the highest prescribers of opioids among all medical specialties, orthopaedic surgeons have the opportunity to have a concerted effect on this crisis.^4^ Orthopaedic surgeons have implemented many successful strategies to reduce opioid prescription and postoperative patient opioid consumption, including preoperative counseling, multimodal pain regimens, and institutional opioid-prescribing protocols.^5,6,7,8,9,10,11,12,13^

Opioid use and dependence have been shown to have numerous deleterious effects on orthopaedic patients. New patients presenting to orthopaedic hand surgery clinics with baseline opioid use have been found to have worse functional outcome scores and increased psychological impairment compared with nonopioid users.^14^ After orthopaedic surgery, patients with prolonged opioid use and dependence have been found to experience poorer surgical outcomes, with lower functional outcome scores and an increased risk for medical complications compared with nonopioid users.^15,16^

The underlying risk factors for orthopaedic surgery patients developing opioid dependence postoperatively have been a focus of many recent studies.^17,18,19,20^ Most studies have investigated patient-specific risk factors such as demographics, comorbidities, and prior substance use. However, few studies have investigated how opioid prescription by the operating surgeon affects prolonged postoperative opioid use, and in those that have, the data are heterogenous and conflicting.^21,22,23,24,25,26,27^

During the perioperative period, patients are at risk for a variety of medical and surgical complications, and it is therefore a vulnerable period for patients. The initial postoperative opioid prescription written by the operating surgeon represents a modifiable risk factor that could affect postoperative patient outcomes. In addition, the initial opioid prescription could be many patients' first exposure to opioids. The purpose of this study was to investigate whether surgeons' prescribing patterns of the initial postoperative opioid prescription predispose patients to prolonged opioid use after hand and upper extremity surgery. We hypothesized that patients prescribed larger initial opioid prescriptions would be more likely to develop prolonged opioid use postoperatively.

## Methods

This was a retrospective study conducted at three urban academic institutions. Institutional review board approval was obtained at all institutions before beginning this study. From April 30, 2018, to August 30, 2019, all patients aged at least 18 years who underwent carpal tunnel release (“CTR,” current procedural terminology [CPT] 64721), basal joint arthroplasty (“BJA,” CPT 25447), and distal radius fracture open reduction and internal fixation (“DRF ORIF,” CPT 25609) performed by 14 board-certified fellowship-trained orthopaedic hand and upper extremity surgeons were collected through database query. This date range was selected to prevent the study period from overlapping with COVID-19 lockdowns, which may not represent typical opioid-prescribing trends. All included patients filled an opioid prescription within 3 days of their surgery date, and only had a single surgery during the study period. The data collected from the three institutions were pooled and analyzed together to diversify surgeons' prescribing patterns and increase the generalizability of results.

Using the Pennsylvania Prescription Drug Monitoring Program (PDMP) website, all opioid prescriptions from 3 months preoperatively to 6 months postoperatively were collected. The Pennsylvania PDMP contains prescription data from 19 states, including Pennsylvania, Arkansas, Connecticut, Delaware, Florida, Louisiana, Maine, Maryland, Massachusetts, Military Health System, Minnesota, New York, North Carolina, Ohio, Oklahoma, Rhode Island, South Carolina, Texas, Virginia, and West Virginia. Specific data collected from the PDMP website include the type of opioid prescribed, prescription strength, prescription date, the number of pills prescribed, the duration of the prescription, and milligram morphine equivalents (MMEs) prescribed. Prescriptions are described in this study by the total MMEs per prescription to standardize the prescription measurement units across different opioid types. The Centers for Disease Control and Prevention lists average daily dose of ≥50 MMEs, initial postoperative opioid prescriptions written for >7 days, and overlapping opioid prescriptions as risk factors for developing opioid dependence, so these variables were included in our analysis.^1^ The primary dependent outcomes evaluated included (1) filling an additional opioid prescription postoperatively and (2) continued postoperative opioid use 3 to 6 months after surgery (“prolonged use”).

Data were first broken down descriptively to understand the distributions of the data. Continuous data are presented as mean (SD), and categorical data are presented as cell count (%). Student *t*-test or Mann-Whitney *U* tests were done to calculate *P* values depending on the normality of the data. Normality was assessed by performing Shapiro-Wilks tests. Chi-square analyses were used to calculate *P* values for categorical data. After this, stepwise logistic regressions were done to identify variables that were significantly associated with the primary outcomes. Regression variables included characteristics of the initial postoperative opioid prescription, including age, sex, preoperative opioid exposure, quantity of pills prescribed, duration of prescription in days, total MME per prescription, prescription written for ≥50 MMEs per day, prescriptions written for >7 days duration, and if the initial postoperative prescription overlapped with an opioid prescription already prescribed to the patient. Significance was determined at *P* value < 0.05. All statistical analyses were done using R Studio (version 4.0.2).

## Results

A total of 634 patients met inclusion criteria, consisting of 276 CTRs, 217 DRF ORIFs, and 141 BJAs. This consisted of 196 men (30.9%) and 438 women (69.1%) at an average age of 59.4 years (SD 14.7 years) (Table 1). Preoperative opioid use was observed in 28.5% of the patients (181/634). The initial postoperative opioid prescription provided by each patient's surgeon was written for an average duration of 4.3 days (SD 3.97), pill quantity of 21.6 pills (SD 14.3), and total MMEs per prescription of 139 MMEs (SD 137). This initial prescription was written for ≥50 MME per day in 18.5% of the patients (117/634), was written for a duration of >7 days in 4.7% of the patients (30/634), and was written overlapping with a concurrently prescribed opioid prescription in 6.3% of the patients (40/634). Postoperatively, 30.1% of the patients (191/634) filled at least one additional opioid prescription, and 14.0% of the patients (89/634) experienced prolonged opioid use 3 to 6 months postoperatively. The 30.1% of the patients who filled an additional opioid prescription postoperatively would fill an average of 2.6 (SD 2.7) additional opioid prescriptions.

**Table 1:** Comparing Risk Factors and Demographic Data of Patients Who Filled Additional Opioid Prescriptions With Those Who Did Not, and of Patients With Prolonged Postoperative Opioid Use With Those Who Did Not

	Total N = 634	Zero Refills N = 443	≥1 Opioid Refill N = 191	*P* Value	No Prolonged Use N = 545	Prolonged 3-6 mo Use N = 89	*P* Value
Age (yr)	59.4 (14.7)	59.2 (15.1)	59.9 (13.6)	0.735	59.2 (14.7)	60.6 (14.7)	0.452
Sex				0.772			0.324
Female	438 (69.1%)	304 (68.6%)	134 (70.2%)		381 (69.9%)	57 (64.0%)	
Male	196 (30.9%)	139 (31.4%)	57 (29.8%)		164 (30.1%)	32 (36.0%)	
Preoperative opioid use				<0.001			<0.001
No	453 (71.5%)	355 (80.1%)	98 (51.3%)		423 (77.6%)	30 (33.7%)	
Yes	181 (28.5%)	88 (19.9%)	93 (48.7%)		122 (22.4%)	59 (66.3%)	
Initial postoperative opioid prescription							
Daily dose ≥ 50 MMEs				0.615			0.571
No	517 (81.5%)	364 (82.2%)	153 (80.1%)		442 (81.1%)	75 (84.3%)	
Yes	117 (18.5%)	79 (17.8%)	38 (19.9%)		103 (18.9%)	14 (15.7%)	
>7 Day duration				0.026			<0.001
No	604 (95.3%)	428 (96.6%)	176 (92.1%)		528 (96.9%)	76 (85.4%)	
Yes	30 (4.73%)	15 (3.39%)	15 (7.85%)		17 (3.12%)	13 (14.6%)	
Overlapping opioid prescriptions				<0.001			<0.001
No	594 (93.7%)	431 (97.3%)	163 (85.3%)		528 (96.9%)	66 (74.2%)	
Yes	40 (6.31%)	12 (2.71%)	28 (14.7%)		17 (3.12%)	23 (25.8%)	
Total MME/prescription	139 (137)	122.6 (77.3)	177.0 (215.2)	0.002	128.7 (81.3)	202.1 (298.2)	0.318
Duration (d)	4.25 (3.97)	3.9 (2.9)	5.1 (5.7)	0.009	3.9 (2.7)	6.4 (7.9)	0.026
Quantity of pills	21.6 (14.3)	19.7 (9.6)	25.9 (21.1)	0.001	20.6 (10.5)	27.4 (27.5)	0.277

MME = milligram morphine equivalent

### Risk Factors for Filling an Additional Opioid Prescription Postoperatively

**Figure 1:**
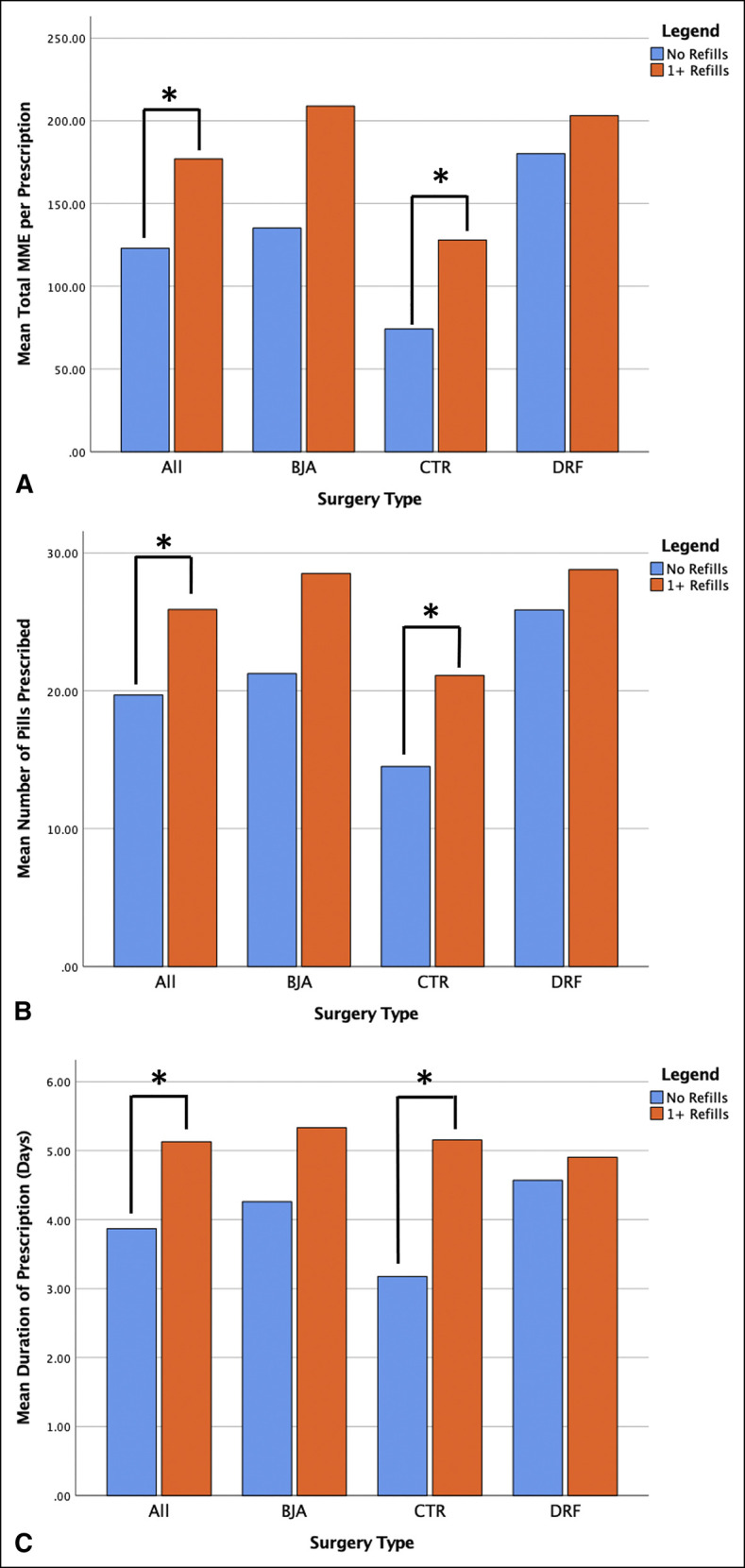
Graphs showing initial opioid prescription characteristics of patients who did and did not fill an additional opioid prescription. **A**, Mean total MME per prescription; **B**, mean number of pills prescribed per prescription; and **C**, mean duration of prescription in days. *Statistically significant difference. MME = milligram morphine equivalent

Patients who filled at least one additional opioid prescription postoperatively were significantly more likely to have been preoperative opioid users (48.7% versus 19.9%, *P* < 0.001), to have been written a prescription for >7 days duration (7.9% versus 3.4%, *P* = 0.026), and to have been written a prescription that overlapped with a previous opioid prescription (14.7% versus 2.7%, *P* < 0.001). In addition, patients who filled an additional prescription postoperatively were initially prescribed significantly more pills (*P* = 0.001), were prescribed a significantly longer duration prescription in days (*P* = 0.009), and were prescribed a significantly larger prescription in total MMEs (*P* = 0.002) than patients who did not fill an additional prescription (Table 1 and Figure 1).

### Risk Factors for Prolonged Opioid Use 3 to 6 Months Postoperatively

**Figure 2:**
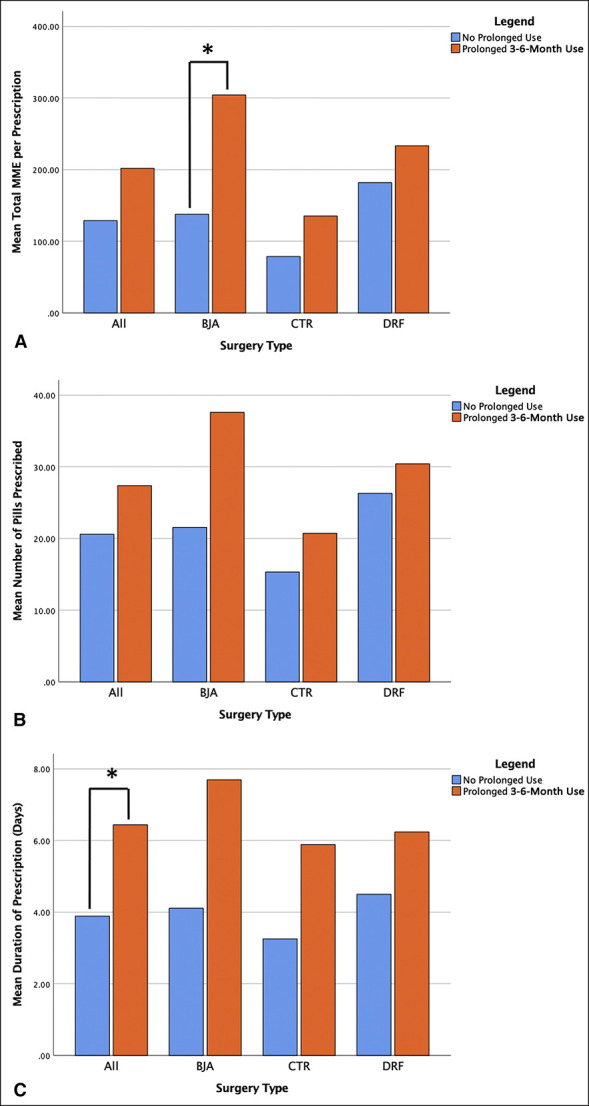
Graphs showing initial opioid prescription characteristics of patients who did and did not experience prolonged 3- to 6-month postoperative opioid use. **A**, Mean total MME per prescription; **B**, mean number of pills prescribed per prescription; and **C**, mean duration of prescription in days. *Statistically significant difference. MME = milligram morphine equivalent

Patients who experienced prolonged opioid use 3 to 6 months postoperatively were significantly more likely to have been preoperative opioid users (66.3% versus 22.4%, *P* < 0.001), to have been written a prescription for >7 days duration (14.6% versus 3.1%, *P* < 0.001), and to have been written a prescription that overlapped with a previous opioid prescription (25.8% versus 3.1%, *P* < 0.001). In addition, patients who had prolonged opioid use postoperatively were prescribed a significantly longer duration prescription in days (*P* = 0.026) compared with those who did not have prolonged postoperative opioid use (Table 1 and Figure 2).

### Multivariable Logistic Regression Analysis

Stepwise logistic regressions were done using filling an additional postoperative opioid prescription and prolonged 3- to 6-month postoperative opioid use as the dependent outcomes. Preoperative opioid exposure (odds ratio [OR] 2.80 [95% confidence interval [CI]: 1.84 to 4.27], *P* < 0.001), greater quantity of pills prescribed (OR 1.04 [95% CI: 1.00 to 1.07], *P* = 0.04), and overlapping opioid prescriptions (OR 2.93 [95% CI: 1.38 to 6.55], *P* < 0.01) were found to be significant predictors of filling an additional opioid prescription postoperatively. Preoperative opioid exposure (OR 5.06 [95% CI: 2.86 to 9.01], *P* < 0.001) and overlapping opioid prescriptions (OR 4.48 [95% CI: 2.09 to 9.85], *P* < 0.001) were found to be significant predictors of prolonged opioid use 3 to 6 months postoperatively. No other variables were found to have statistically significant associations with either outcome analyzed.

### Results by Procedure Type

When investigating the outcomes of CTR, BJA, and DRF ORIF individually, patients who filled additional opioid prescriptions and patients with prolonged opioid use were both found to have been prescribed larger initial prescriptions in total MMEs, more pills per prescription, and longer duration prescriptions in days for each procedure (Figures 1 and 2). Table 2 details the characteristics of the initial postoperative opioid prescription for the three procedures included in this study.

**Table 2:** Initial Postoperative Opioid Prescription Characteristics Between the Included Procedure Types

	BJA N = 141	CTR N = 276	DRF ORIF N = 217
Sex			
Female	105 (74.5%)	184 (66.7%)	149 (68.7%)
Male	36 (25.5%)	92 (33.3%)	68 (31.3%)
Age (yr)	62.8 (9.15)	59.7 (15.2)	56.8 (16.4)
Total MME/Rx	165 (189)	88.1 (115)	187 (93.4)
Duration (d)	4.70 (5.17)	3.68 (3.95)	4.67 (2.87)
Quantity of pills	24.2 (20.0)	16.2 (11.8)	26.7 (9.84)

BJA = basal joint arthroplasty, CTR = carpal tunnel release, DRF ORIF = distal radius fracture open reduction and internal fixation, MME = milligram morphine equivalent, Rx = prescription

## Discussion

For many orthopaedic hand and upper extremity procedures, postoperative opioid prescriptions are prescribed for patient comfort and satisfaction during the postoperative period. However, the use of opioids in itself can place patients at risk for a multitude of adverse outcomes postoperatively, including opioid dependence, poorer functional outcomes, and medical complications.^14-16,18,28^ This study revealed that characteristics of the initial postoperative opioid prescription written by the operating surgeon could predispose patients to filling additional opioid prescriptions and prolonged opioid use postoperatively. Specifically, overlapping opioid prescriptions, longer duration prescriptions, prescriptions with a larger number of pills, and larger prescriptions in total MMEs were markedly associated with persistent opioid use postoperatively. These findings could represent potentially modifiable risk factors for orthopaedic surgeons to help prevent the development of persistent opioid use in their patients after hand and upper extremity surgery.

Numerous previous studies have evaluated risk factors for prolonged opioid use after orthopaedic surgery; however, most of these studies focus on patient-specific risk factors, such as medical comorbidities and prior opioid use.^17,18,19,20^ In hand surgery specifically, studies have identified preoperative opioid use, alcohol use, smoking history, younger age, lower income, psychiatric disorders, benzodiazepine use, and higher comorbidity scores as risk factors for prolonged postoperative opioid use.^17,18,28^

However, very few studies could be identified that investigate how opioid-prescribing patterns of the operating surgeon may contribute to prolonged opioid use. Hozack et al^25^ performed a retrospective study of state PDMP data for 290 patients undergoing a wide variety of hand and upper extremity surgeries to investigate whether the amount of opioids prescribed on the day of surgery correlated with prolonged use postoperatively. They concluded that neither the amount of opioids provided intraoperatively, the amount provided in the recovery room, nor the amount prescribed on the day of surgery correlated with the number of opioid prescriptions filled within 6 months postoperatively. Delaney et al^22^ used a Medicare claims database to examine operating surgeons' prescribing patterns and new prolonged opioid use in opioid naïve patients undergoing total hip arthroplasty. The authors divided surgeons into quartiles based on initial prescription size and investigated other high-risk prescribing factors such as high daily doses, overlapping benzodiazepine prescriptions, overlapping opioid prescriptions, receiving prescriptions from multiple providers, and the use of long acting opioids. They determined that patients of surgeons who exhibited the highest rates of high-risk opioid prescribing were significantly more likely to develop new prolonged opioid use postoperatively (9.7% versus 4.6%, *P* = 0.011). In addition, patients of surgeons who wrote the largest prescription sizes were at a three times higher risk for prolonged opioid use postoperatively (OR 2.91; 95% CI, 1.53 to 5.51). Within a much shorter follow-up period of 10 to 14 days after minor hand surgery, Gaddis et al^23^ observed no difference in the number of refills requested between patients who received 10 pills of hydrocodone/acetaminophen versus 30 pills on their surgery date. However, the 2-week maximum follow-up in this study likely would not accurately capture patients who eventually develop prolonged opioid dependence. Multiple large database studies consisting of both surgical and nonsurgical opioid naïve patients with noncancer pain have shown that larger index opioid prescriptions are associated with patients developing long-term opioid dependence.^29,30^ Overall, the currently available data on this topic are very heterogenous, and the results are conflicting.^21,22,23,24,25,26,28^

It has been well established that opioids are overprescribed in orthopaedic surgery, and especially in hand surgery.^8,23,31,32^ In addition, studies have shown that patients take more opioids when they are given larger prescriptions, although for some hand procedures, many patients achieve adequate pain control without the use of any opioids.^13,23,27,32,33^ Gaddis et al^23^ performed a prospective randomized study of patients undergoing minor hand surgery being provided 10 or 30 opioid pills postoperatively. They observed that patients who received larger prescriptions consumed markedly more pills after surgery and were markedly more likely to still be taking opioids at the 2-week follow-up visit (15% versus 4%). Given these findings and the results of our study, it is clear that surgeons' prescribing patterns of the initial postoperative opioid prescription could predispose patients to developing prolonged opioid dependence after surgery. Opioid-prescribing patterns of surgeons are likely multifactorial, with residency/fellowship training experience, time in practice, and personal experiences all contributing. Patient characteristics may also play a role in postoperative opioid prescribing, with one study finding that factors such as patient sex, race, insurance type, history of chronic pain, and history of illicit substance use may contribute to the amount of opioids prescribed after hand surgery.^34^

In hand and upper extremity surgery, researchers have proposed the opioid-prescribing guidelines to address these variations in opioid-prescribing patterns among orthopaedic surgeons. Kim et al^8^ prospectively evaluated opioid consumption patterns of patients undergoing a variety of hand and upper extremity surgeries in the acute postoperative period. Based on their findings, they recommended upper extremity surgeons prescribe ≤10 opioid pills for hand/wrist soft-tissue procedures, ≤15 pills for elbow/forearm soft-tissue procedures, ≤20 pills for hand/wrist/elbow/forearm fracture or joint procedures, and ≤30 pills for upper arm/shoulder procedures. At the systems' level, some centers have implemented institutional protocols and educational resources for surgeons on postoperative opioid prescribing. These interventions have been shown to markedly decrease opioid prescription sizes among participating surgeons.^10,11^ Some have even described successfully eliminating any and all opioid prescription in their hand and upper extremity surgery practice.^33^

This study has several strengths. It includes data from 14 orthopaedic surgeons from three academic institutions which each care for different patient populations, which increases the generalizability of our findings. Previous studies have included patients undergoing dozens of different hand surgical procedures, which could introduce bias between study groups. To combat this, we included only three common hand and upper extremity procedures, which limits the procedural variation of previous studies. This study also has several weaknesses. Our dependent outcomes were based on filled opioid prescriptions, which may not equate to actual opioid consumption. Much of the data in this study were obtained from the state PDMP system website, which does not include any information on the indications for patients receiving postoperative refills. Similarly, as a retrospective study, we are unable to ascertain the indications for patients filling opioid prescriptions preoperatively. It is possible that some patients used opioids chronically; however, the variable of preoperative opioid use is controlled for through its inclusion in the multivariable logistic regression model. It is possible that patients filled opioid prescriptions in states outside of the 19 available for access in this study, although we believe this would be unlikely. As all data were obtained retrospectively, we are unable to know if patients used nonopioid analgesics such as acetaminophen and NSAIDs in addition to opioids during postoperative recovery.

The initial postoperative opioid prescription written by the operating surgeon must ensure adequate pain control, meanwhile avoiding overprescribing, which could potentially result in opioid misuse and diversion. This study found that larger initial prescriptions, in both pill quantity and total MMEs, as well as longer duration prescriptions were notable predictors of patients filling additional opioid prescriptions and developing prolonged opioid use after hand and upper extremity surgery. These findings emphasize the importance of safe and evidence-based prescribing practices, including using PDMP databases, to prevent the detrimental effects of opioid use postoperatively. We recommend surgeons adopt and use strategies that have been proven to decrease postoperative opioid use, including preoperative patient counseling, multimodal pain regimens, and opioid-prescribing protocols for specific procedures.
